# Econazole nitrate inhibits PI3K activity and promotes apoptosis in lung cancer cells

**DOI:** 10.1038/s41598-017-18178-0

**Published:** 2017-12-21

**Authors:** Chao Dong, Runxiang Yang, Hongjian Li, Kunbin Ke, Chunxiang Luo, Fang Yang, Xi-Nan Shi, Ying Zhu, Xu Liu, Man-Hon Wong, Guimiao Lin, Xiaomei Wang, Kwong-Sak Leung, Hsiang-Fu Kung, Ceshi Chen, Marie Chia-mi Lin

**Affiliations:** 1 Biomedical Engineering Research Center, Kunming Medical University, Kunming, Yunnan China; 2 Department of the second medical oncology, The 3rd Affiliated Hospital of Kunming Medical University, Yunnan Tumor Hospital, Kunming, China; 3 Institute of Future Cities, Chinese University of Hong Kong, Hong Kong, China; 4 Department of Computer Science and Engineering, Chinese University of Hong Kong, Hong Kong, China; 5 Department of Urology, the 1st Affiliated Hospital of Kunming Medical University, Kunming, China; 6 School of Basic Medical Sciences, Shenzhen University Health Sciences Center, Shenzhen, China; 7 Key Laboratory of Animal Models and Human Disease Mechanisms of Chinese Academy of Sciences and Yunnan Province, Kunming Institute of Zoology, Chinese Academy of Sciences, Kunming, Yunnan China; 8 Academy of Medical Science, Zhengzhou University, Zhengzhou, Henan Province, China

## Abstract

The phosphatidylinositol-3-kinase (PI3K)/AKT signaling pathway plays a pivotal role in many cellular processes, including the proliferation, survival and differentiation of lung cancer cells. Thus, PI3K is a promising therapeutic target for lung cancer treatment. In this study, we applied free and open-source protein-ligand docking software, screened 3167 FDA-approved small molecules, and identified putative PI3Kα inhibitors. Among them, econazole nitrate, an antifungal agent, exhibited the highest activity in decreasing cell viability in pathological types of NSCLC cell lines, including H661 (large cell lung cancer) and A549 (adenocarcinoma). Econazole decreased the protein levels of p-AKT and Bcl-2, but had no effect on the phosphorylation level of ERK. It inhibited cell growth and promote apoptosis in a dose-dependent manner. Furthermore, the combination of econazole and cisplatin exhibited additive and synergistic effects in the H661 and A549 lung cancer cell lines, respectively. Finally, we demonstrated that econazole significantly suppressed A549 tumor growth in nude mice. Our findings suggest that econazole is a new PI3K inhibitor and a potential drug that can be used in lung cancer treatment alone or in combination with cisplatin.

## Introduction

Lung cancer is the most common malignancy worldwide and the leading cause of cancer-related death, with 1.6 million lives each year attributable to the disease (according to WHO 2012 estimates). Non-small-cell lung cancer (NSCLC) is the most common form of lung cancer, accounting for 85% of all cases^1^. The traditional treatments for NSCLC include surgery, chemotherapy and radiotherapy, but the five-year survival rate is only 15%. In recent decades, targeted therapies against EGFR mutations and ALK rearrangements have improved patient prognoses. However, new therapeutic targets and drugs are urgently needed for lung cancer treatment.

The phosphatidylinositol 3-kinase (PI3K)/AKT signaling pathway promotes carcinogenesis and the development of a variety of human cancers, including NSCLC^2,3^. Genetic alterations of the PI3K pathway, such as PIK3CA mutation and PTEN mutation and loss, are observed in 16% of NSCLC cases^4^. Yamamoto *et al*. identified PIK3CA mutations in 4.7% of more than 700 lung cancer samples^5^. In another study, 3% (34/1117) of NSCLC patients harbored PIK3CA mutations, and no significant correlation between PIK3CA mutations and pathological types was detected^6^. The PIK3CA gene encodes the p110α catalytic subunit of PI3K^7,8^. In one study, high cytoplasmic PI3K p110α expression was detected in 48.6% (69/142) of NSCLC samples regardless of the mutation status of PIK3CA^6^. As a central node in this pathway, PI3K represents an attractive therapeutic target for lung cancer treatment.

Currently, several PI3K inhibitors, including the pan-PI3K inhibitor buparlisib (BKM120) and the PI3Kα-selective inhibitor alpelisib (BYL719), are being evaluated in clinical trials^9–11^. The phase II BASALT-1 study reported that the 63 NSCLC patients who received single-agent buparlisib (100 mg/day) achieved good clinical outcomes^12^. Combinations of PI3K inhibitors with other agents, such as doxorubicin, dichloroacetate, docetaxel, and temozolomide, exhibit better efficacy than monotherapy^10,13,14^.

Computer-aided drug design has become an important method for drug development, which has greatly accelerated the efficiency of drug discovery and reduced costs. Molecular docking, as the core technology, is a computational method in which small molecule ligands are docked to the active pockets of the receptor (the target protein) to predict candidate drugs. Using this approach, our team has previously identified fluspirilene as a potential CDK2 inhibitor^15^.

Among the catalytic subunits of PI3K, PI3Kα is closely associated with tumorigenesis. Therefore, PI3Kα appears to be an ideal target for drug development. In this study, we utilized free and open-source protein-ligand docking software idock^16,17^, binding affinity prediction software RF-Score-v3^18^, and molecular visualization tool iview^19^ to screen 3167 FDA-approved small molecules to identify putative PI3Kα inhibitors. Among them, econazole nitrate, an antifungal agent, exhibited the highest activity in decreasing cell viability in pathological types of NSCLC cell lines, including H661 (large cell lung cancer), A549 (adenocarcinoma), NCI-H520 and SK-SEM-1 (squamous cell carcinoma), but had little cytotoxicity in BEAS-2B (normal lung bronchial epithelial cell line). In silico, we analyzed the econazole binding conformation and recognized potential critical intermolecular interactions between econazole and PI3Kα. Compared to the known PI3K inhibitors (BLY719 and BKM120), econazole showed the more potent cytotoxicity in H661 cells. Econazole decreased the protein levels of p-AKT and Bcl-2, but had no effect on the phosphorylation level of ERK. As expected, econazole inhibited cell growth and induced apoptosis in the H661 and A549 lung cancer cell lines in a dose-dependent manner. Furthermore, the combination of econazole and cisplatin exhibited synergistic and additive effects in H661 and A549 lung cancer cells, respectively. Furthermore Econazole significantly suppressed A549 tumor growth in nude mice. Our findings suggest that econazole is a novel PI3K inhibitor and a potential drug that can be used in lung cancer treatment alone or in combination with cisplatin.

## Results

### Ensemble docking, drug repositioning, and compound selection

In total, 3167 FDA-approved drugs were docked and ranked according to their average predicted binding affinity across 8 X-ray crystal structures of PI3Kα. The drugs were individually docked to the ATP binding pocket of PI3Kα and then sorted in ascending order of their predicted binding free energy. The visual docking results using the 4JPS entry are available at http://istar.cse.cuhk.edu.hk/idock/iview/?4JPS-dbap+fda+npc. Eight compounds (Table 1) were selected for subsequent investigation.

**Table 1:** Characteristics of the eight high-scoring compounds tested.

ZINC ID	idock score	RF score	Name	Clinical usage
596881	−7.30	7.07	Econazole nitrate	Antifungal
3810860	−8.78	7.09	Ezetimibe	Cholesterol absorption inhibitor
601275	−8.97	7.15	Talniflumate	Anti-inflammatory and analgesic effect
3802417	−8.14	7.24	Alvimopan	Treatment of gastrointestinal dysfunction
13831810	−8.01	7.67	Mizolastine	H1 receptor antagonists
29489118	−8.52	7.11	Flupentixol dihydrochloride	Antidepressants
601242	−8.16	7.30	doxazosin mesylate	Treatment of hypertension
11677837	−8.11	7.57	Apixaban	Thrombosis prevention

Compounds with a low idock score (a measurement of binding free energy in kcal/mol units) and a high RF score (a measurement of binding affinity in pKd units) may likely bind the PI3Kα protein.

### Econazole inhibits lung cancer cell viability

**Figure 1:**
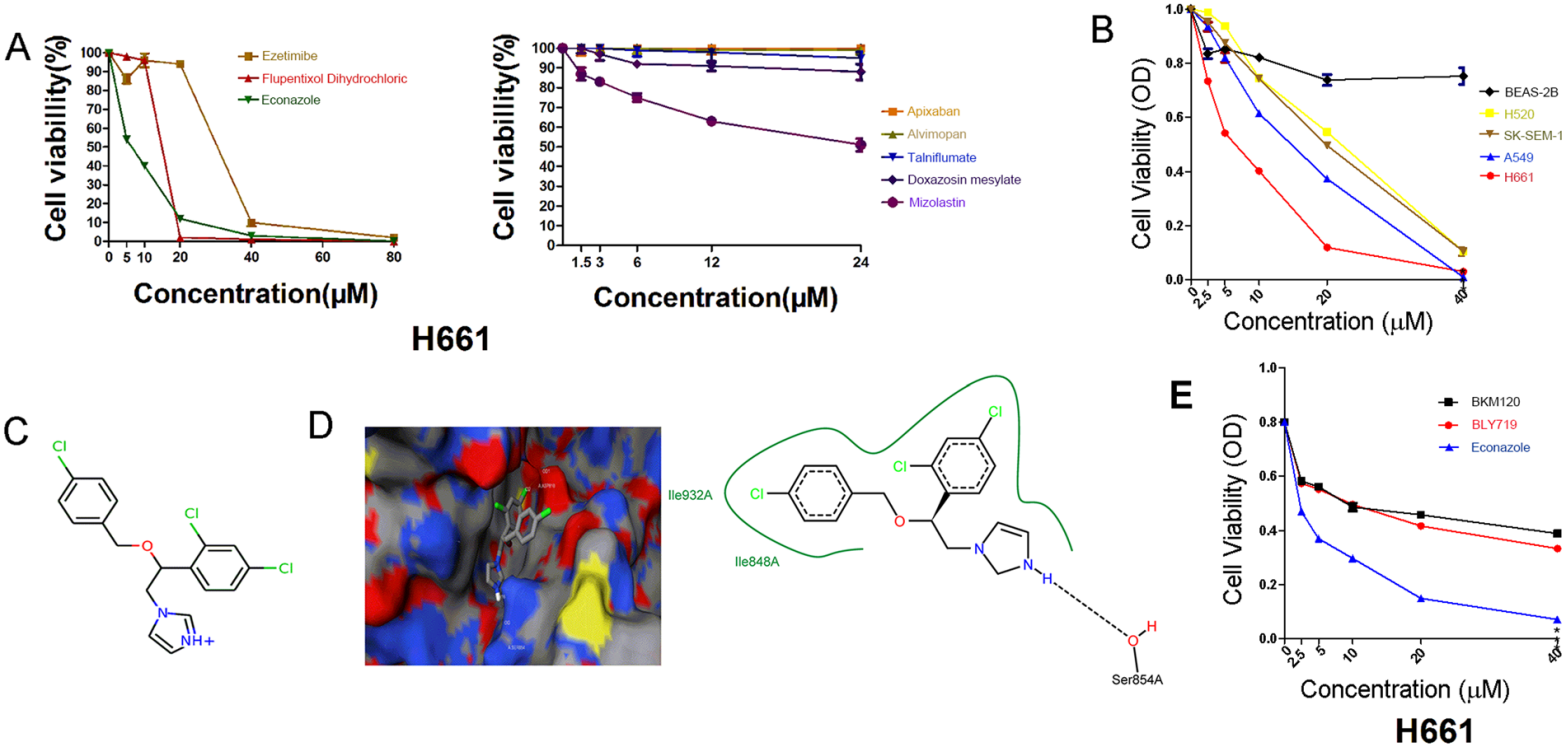
Putative PI3Kα inhibitors suppress lung cancer cell line viability. (**A**) Eight candidate PI3Kα inhibitors decreased the viability of H661 lung cancer cells with discrepant cytotoxicity at different concentrations. (**B**) Econazole (2.5, 5, 10, 20, or 40 μM) inhibited the viability of four NSCLC cell lines (H661, A549, H520 and SK-SEM-1) in a dose-dependent manner, but has little effect on BEAS-2B cells. The cells were treated with econazole for 24 hours. (**C**) The structure of econazole. (**D**) The predicted conformation of econazole in complex with PI3Kα (PDB ID: 4JPS). PI3Kα is depicted as a molecular surface representation colored by atom type. Econazole is rendered as sticks colored by atom type. Intermolecular interacting atoms and residues are labeled. The cyan and wheat dashed lines indicate a hydrogen bond and a halogen bond, respectively. This figure was created using the web-based visualizer iview. (**E**) The 24-hour cytotoxicity of econazole and two known PI3K inhibitors (PI3Kα-selective inhibitor BLY719 and pan-PI3K inhibitor BKM120) in H661 cells.

We tested the anticancer effects of the eight compounds using the MTT assay. Five of the eight compounds decreased cell viability in the H661 lung cancer cell line but exhibited discrepant cytotoxicity at different concentrations (Fig. 1A). Among them, econazole (ZINC ID 00596881) exhibited the strongest anticancer effect in a dose-dependent manner (IC_50_ = 6.0 μM). Next, we tested the inhibitory effects of econazole in other NSCLC cell lines and BEAS-2B, an SV40 immortalized normal lung bronchial epithelial cell line. We observed that econazole exhibited excellent inhibitory effects in multiple NSCLC cell lines in a dose-dependent manner (IC_50_ = 13.5 μM in A549, 22.2 μM in SK-SEM-1, and 22.3 μM in NCI-H520) but had little cytotoxicity in BEAS-2B cells (Fig. 1B). The predicted binding free energy of econazole by idock was −7.3 kcal/mol, and its predicted binding affinity by RF-Score was 7.07 pKd. The 2D structure of econazole is presented in Fig. 1C. The predicted conformation of econazole in complex with PI3Kα protein is shown in Fig. 1D. Putatively, econazole binds to PI3Kα by forming a hydrogen bond with Ser854 and a halogen bond with Asp810 and hydrophobic contacts with Ile848 and Ile932. To test whether the anti-cancer effect of econazole in lung cancer is as potent as other PI3K inhibitors, we compared its cytotoxicity with two known PI3K inhibitors (PI3Kα-selective inhibitor alpelisib BLY719 and pan-PI3K inhibitor buparlisib BKM120) in H661 cells. As shown in Fig. 1E, econazole (IC_50_ = 6.0 μM) was more potent than BLY719 (IC_50_ = 45.9 μM) and BKM120 (IC_50_ = 63.9 μM) in terms of cytotoxicity for 24 hours.

### Econazole specifically inhibits AKT phosphorylation and Bcl-2 gene expression

**Figure 2:**
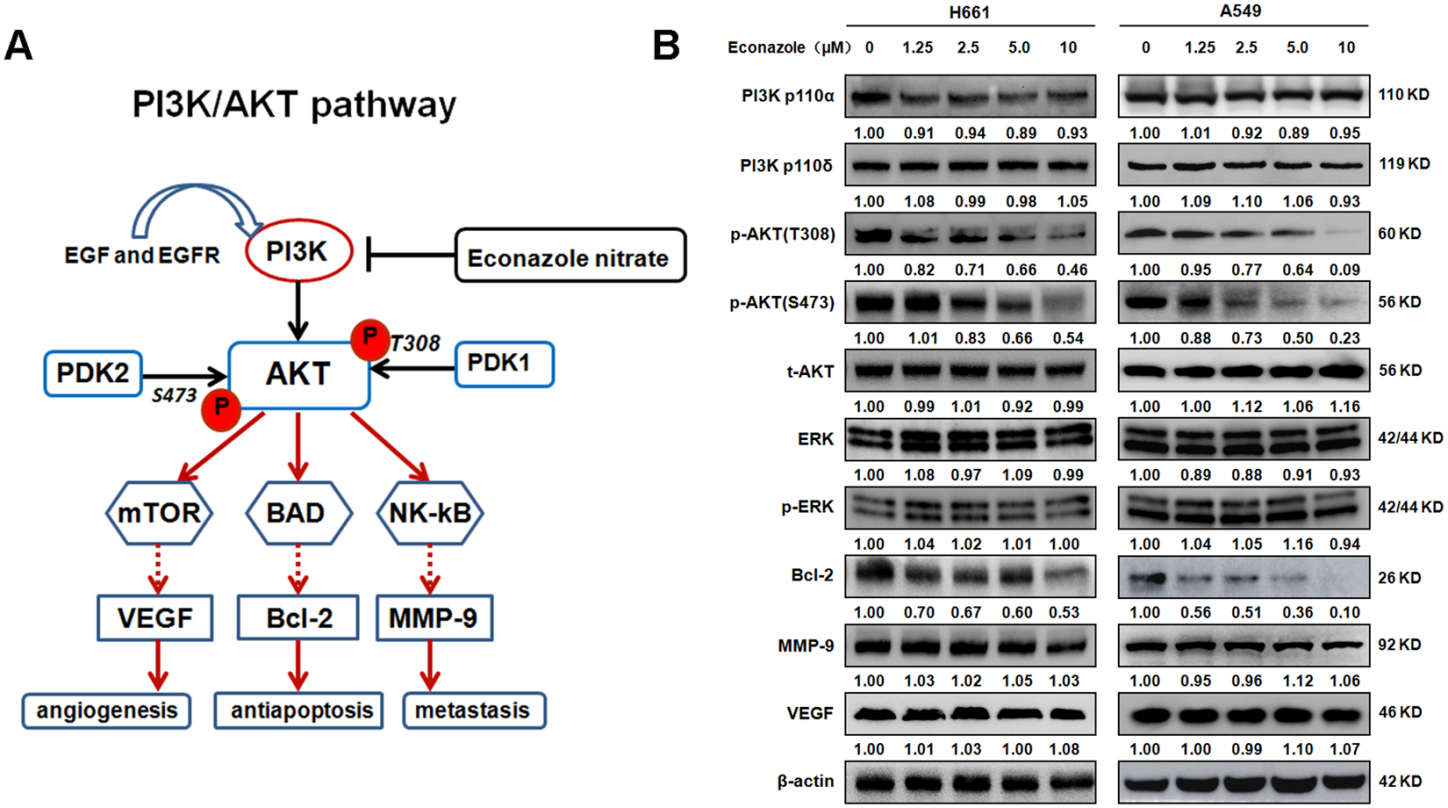
Econazole specifically inhibits the PI3K/AKT pathway in lung cancer cell lines. (**A**) The putative PI3K/AKT signaling pathway. (**B**) H661 and A549 cells were treated with increasing concentrations of econazole (1.25, 2.5, 5, or 10 μM) for 24 hours. The expression levels of p-AKT (S473 and T308) and Bcl-2 were decreased by econazole treatment in a dose-dependent manner. The expression of PI3K p110α in H661 but not A549 cells was decreased by econazole treatment, and econazole had no effect on PI3K p110δ in both cells. The expression levels of t-AKT, ERK, p-ERK, VEGF and MMP-9 were not changed by econazole treatment. Normalized band intensities were added below each band.

The putative PI3K signal transduction pathway is shown in Fig. 2A. The activation of PI3K downstream target AKT phosphorylation plays a pivotal role in enhancing tumor angiogenesis, cell growth/apoptosis, invasion/metastasis in PI3K/AKT pathway. We investigated the effect of econazole on the expression of critical PI3K/AKT signaling pathway proteins by Western blotting (Fig. 2B). H661 and A549 cells were treated with increasing concentrations of econazole for 24 hours. As shown in Fig. 2B, econazole decreased the expression of PI3K p110α in H661 but not A549 cells and had no effect on PI3K p110δ. As expected, AKT phosphorylation (Thr308 and Ser473) levels were significantly decreased by econazole in a dose-dependent manner (>1.25 μM), whereas total AKT protein levels were not affected. Econazole did not decrease the phosphorylation levels of ERK, indicating the specificity of econazole to the PI3K pathway. Econazole also specifically decreased the expression levels of Bcl-2 but not MMP-9 or VEGF, suggesting that econazole induces apoptosis in lung cancer cells.

### Econazole promotes apoptosis in lung cancer cells

**Figure 3:**
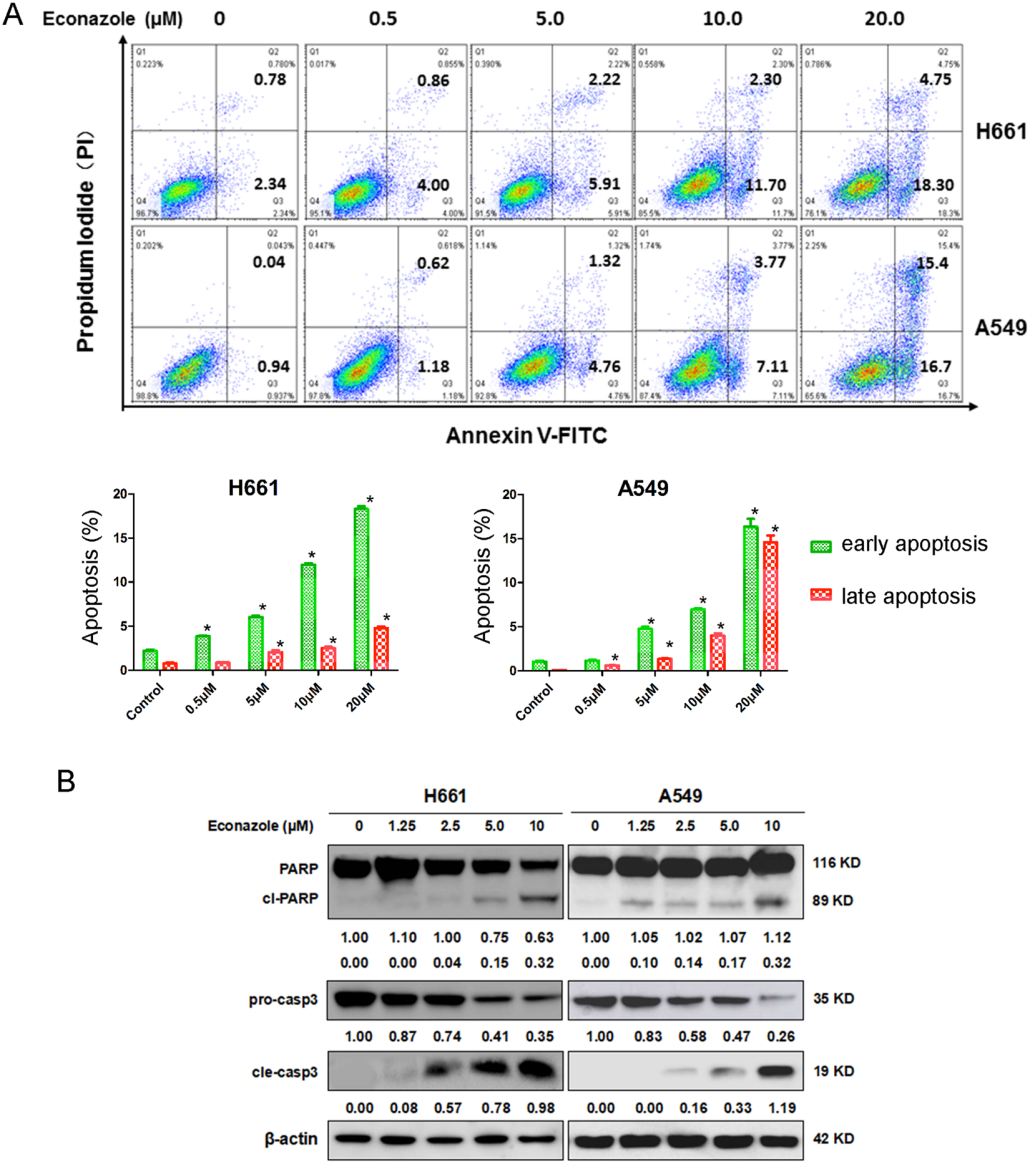
Econazole induces apoptosis in lung cancer cell lines. (**A**) Econazole induced apoptosis in H661 and A549 lung cancer cells. H661 and A549 cells were treated with econazole at 0.5, 5, 10, or 20 μM for 24 hours. Apoptosis was measured by Annexin V/PI staining. (**B**) Econazole induced the cleavage of Caspase-3 and PARP in lung cancer cells, as measured by Western blotting. H661 and A549 cells were treated with econazole (1.25, 2.5, 5, or 10 μM) for 24 hours. Normalized band intensities were added below cleaved PARP and Caspase-3.

We treated H661 and A549 cells with econazole (0.5, 5, 10 and 20 μM) for 24 hours and performed Annexin V staining. As shown in Fig. 3A and B, econazole significantly and dose-dependently increased the percentage of Annexin V-positive H661 and A549 cells. To test whether econazole activates apoptotic signaling in lung cancer cells, we examined the cleavage of poly-(ADP-ribose)-polymerase (PARP) and caspase-3, two apoptotic biomarkers. Both H661 and A549 cells were treated with varied concentrations of econazole (0, 0.5, 5, 10, or 20 μM) for 24 hours. Econazole induced PARP and caspase-3 cleavage in a dose-dependent manner (Fig. 3B). These results demonstrated that econazole promotes apoptosis in lung cancer cells.

### Combination of econazole and cisplatin produced additive effect in lung cancer cells

**Figure 4:**
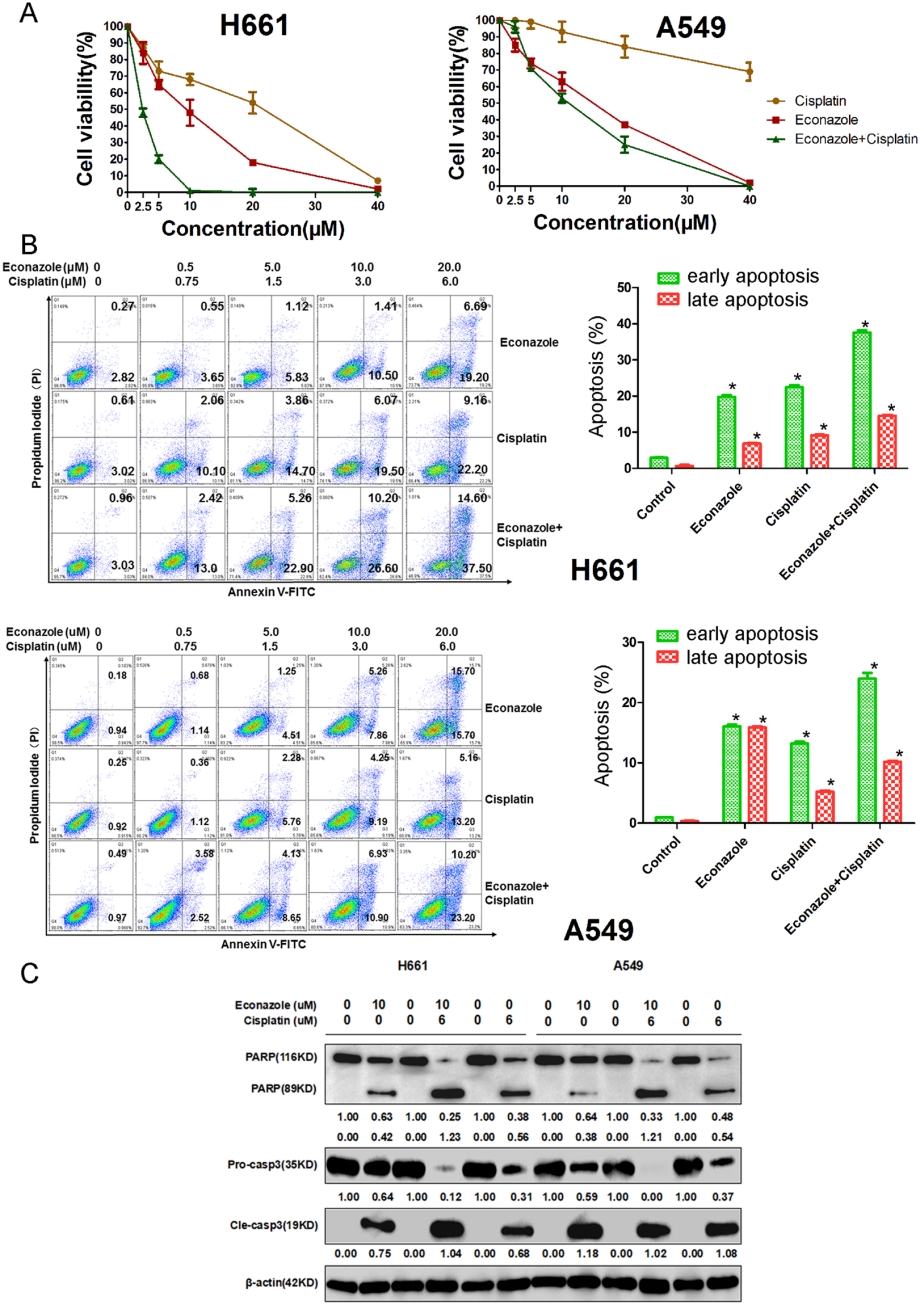
Econazole and cisplatin additively inhibited lung cancer cell line viability. (**A**) Econazole and cisplatin additively decreased the cell viability of H661 and A549 cells. The combination of econazole and cisplatin exhibited stronger inhibition than did any single drug. (**B**) Econazole and cisplatin additively induced apoptosis in H661 and A549 lung cancer cells. The combinations of econazole with cisplatin at the varying concentrations significantly induced more apoptosis in H661 and A549 cells than any single drug did, as measured by Annexin V/PI staining. (**C**) Econazole and cisplatin additively induced greater cleavage of PARP and Caspase-3 than any single drug did, as measured by Western blotting analysis. Normalized band intensities were added below cleaved PARP and Caspase-3.

To evaluate the combination effect of econazole and cisplatin, we treated H661 and A549 cells with increasing concentrations of econazole and cisplatin either alone or in combination for 36 hours. As shown in Fig. 4A, the combination treatment exhibited synergistic and additive killing effects in H661 and A549 cells, respectively, with H661 cells more sensitive to the drug treatments. In addition, we also treated H661 and A549 cells with econazole and cisplatin for 12 hours and measured apoptosis by Annexin V-FITC and propidium iodide (PI) staining, followed by flow cytometry analysis. Results obtained indicated that combination of econazole with cisplatin significantly increased the percentage of apoptotic cells as compared to either econazole or cisplatin alone (Fig. 4B). Consistently, the combination treatment also induced more PARP and caspase-3 cleavage than single drug treatments (Fig. 4C).

### Econazole suppressed A549 tumor growth in nude mice

**Figure 5:**
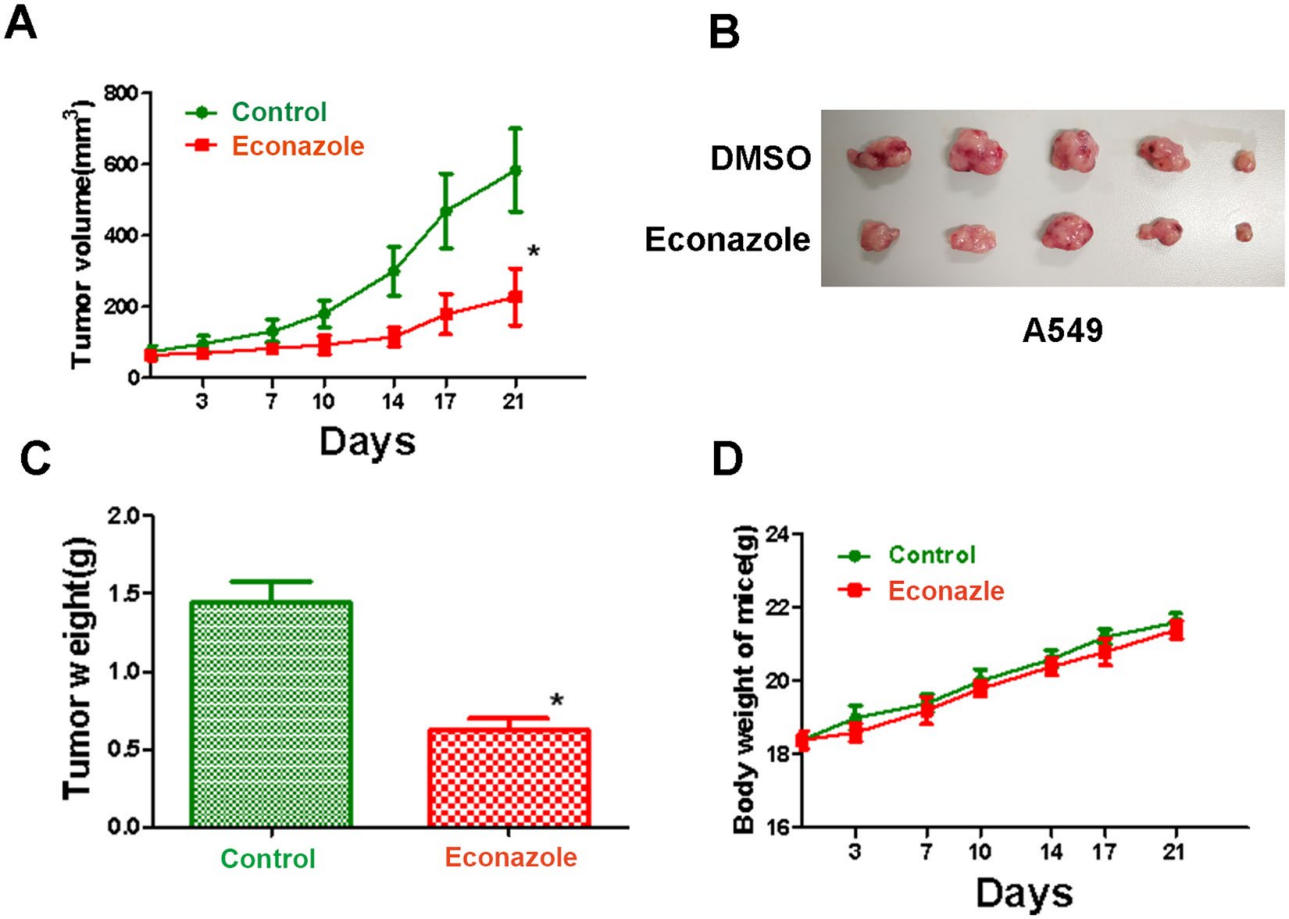
Econazole suppressed A549 tumor growth in nude mice. (**A**) Econazole (50 mg/kg daily) significantly inhibited A549 tumor growth in nude mice. A549 cells were subcutaneously injected in BALB/C nude mice to allow tumors grow to about 70–80 mm^3^. Econazole was administrated I.P. for 21 days. (**B**) The tumor mass harvest at the end of the experiment. (**C**) The tumor weight from the econzaole treatment group and the negative control group. (**D**) The mouse bodyweights at the different time points from the econzaole treatment group and the negative control group.

To evaluate the anti-tumor effect of Econazole on the growth of lung carcinoma *in vivo*, we subcutaneously injected A549 cells in BALB/C nude mice. When tumors grow to about 70–80 mm^3^ (day 7), econazole (50 mg/kg daily) was administrated I.P. for 21 days. As shown in Fig. 5A, econazole significantly suppressed tumor growth compared to the vehicle control. The tumor weight from the econzaole treatment group was also significantly lighter than the negative control group while it did not affect mouse bodyweight. These results suggested that econazole is a candidate anti-cancer drug for human lung adenocarcinoma.

## Discussion

Lung cancer is recognized as the most prevalent type of cancer and is responsible for significant patient mortality. Lung cancers are generally divided into two main categories: small cell lung cancer (SCLC) and non-small cell lung cancer (NSCLC). NSCLC is the most common form of lung cancer, representing 80–85% of all cases. NSCLC is further divided into squamous cell carcinoma (25–30%), adenocarcinoma (35–40%), and undifferentiated large cell carcinoma (10–15%). In recent years, the curative efficacy of traditional treatments, including chemotherapy and radiotherapy, has reached a bottleneck. The toxicity and side effects of chemotherapy and radiotherapy limit their clinical applications. Targeted therapy has become one of the most attractive and promising therapeutic approaches because of its reliable efficacy and mild side effects. The identification of therapeutic driver mutations, such as activating EGFR mutations and rearrangements of the ALK oncogene, has improved the outcomes of patients with these genetically defined NSCLCs within the last decade^20,21^. Thus, it is important to identify more therapeutic targets and their inhibitors for the treatment of lung cancer^22^.

The dysregulation of the PI3K signaling pathway is closely related to tumor development, metastasis, apoptosis and drug resistance in lung cancer^23^. PI3K belongs to a large family of lipid kinases that regulate numerous biological processes by generating lipid second messengers^24^. Upon activation, PI3K leads to the phosphorylation of Akt, which promotes downstream signaling proteins involved in cell proliferation/survival, angiogenesis, motility/invasion^25^. AKT is activated by phosphorylation at both Thr-308 and Ser-473 sites^26^, which activates downstream proteins to promote resistance to the EGFR inhibitor gefitinib in lung cancer patients^27,28^. The Bcl-2 family member proteins are crucial PI3K/AKT downstream proteins promoting cell survival. Down-regulation of p-AKT decreases the expression of Bcl-2 in NSCLC^29,30^. Caspase-3, a major executioner caspase in apoptosis, is one of the most important members of the caspase protein family. Caspase-3 is responsible for the downstream execution steps of apoptosis by cleaving PARP^31^. Accumulated reports have indicated that inhibition of AKT phosphorylation simultaneously activates caspase-3 in lung cancer A549 cells^32^, and promotes caspase-3 resistance to EGFR inhibitor therapy in several NSCLC cell lines^28^.

More than twenty PI3K inhibitors have demonstrated favorable anticancer effects in preclinical experiments and have entered clinical trials^33^. Currently, the methods of identifying PI3K inhibitors require advanced techniques and a significant amount of time and money. In this study, we took advantage of in silico tools. With the availability of crystal structures of PI3Kα, we applied free and open-source protein-ligand docking software, and screened 3167 FDA-approved drugs in a short time. We identified new anti-cancer indications of existing drugs that have been previously approved for the treatment of other diseases. Among the eight candidates shortlisted from the virtual screening results, econazole exhibited the strongest anticancer activity. Econazole decreased cell viability in all pathological types of NSCLC cell lines, including H661 (large cell lung cancer), A549 (adenocarcinoma), NCI-H520 and SK-SEM-1 (squamous cell carcinoma). Among the four cell lines, econazole exhibited the strongest inhibitory effect particularly in H661 and A549 cells. Importantly, econazole has little toxicity in normal lung bronchial epithelial cells. Compared to two known PI3K inhibitors, BLY791 and BKM120, econazole showed more potent cytotoxicity in H661.

Molecular docking indicated that econazole binds to the ATP-binding pocket of PI3K through a hydrogen bond and a halogen bond. Computer-aided drug design might provide new ideas for the development of PI3K inhibitors. However, it remains necessary to confirm the drug effects through biological experiments.

Econazole nitrate, one of the imidazole anti-fungal agents, has been used in the treatment of mycosis with a minimum inhibitory dose of 1 mol/m^3^ ^34^. The primary pharmacological structure of econazole is an imidazole ring similar to the structure of PI3K inhibitors. All small molecule PI3K inhibitors competitively bind to the kinase catalytic domain of the ATP-binding pocket, including imidazole, pyridine, quinazoline derivatives, thiazole and other compounds. Therefore, these inferred that the structure of the imidazole ring of econazole may play an important role in the inhibition of PI3Kα. Recent studies demonstrated that econazole exhibits cytotoxic activities in cancer cell lines, although the exact mechanism remains unclear^35,36^. Econazole inhibits the proliferation of the MCF-7 breast cancer cell line *in vitro* and *in vivo* ^35,37,38^.

Similarly, econazole (1–30 μM) inhibits the proliferation of the PC3 prostate cancer cell line by stimulating Ca^2+^ influx into cells^39^. Additionally, econazole (5–20 μM) arrests human colon cancer cells at the G0/G1 phase of the cell cycle^40^. Here, we demonstrated that in lung cancer cell lines, econazole promote apoptosis through inhibiting the activity of PI3Kα via AKT and Bcl2.

Activation of AKT phosphorylation has been implicated as an important signaling pathway for cell survival and apoptosis. Itraconazole, another traditional antifungal drug, has been identified as a novel potential anticancer agent in glioblastoma^41^. Itraconazole induced autophagy and inhibited cell proliferation by repression of PI3K-AKT-mTOR signaling^41–43^.

Bcl-2 is a critical anti-apoptotic protein^44^. Econazole decreased AKT phosphorylation and Bcl-2 protein expression in both H661 and A549 cell lines. Consequently, econazole induces apoptosis, as evidenced by the cleavage of caspase-3 and PARP. The anti-cancer effect of econazole in lung cancer cells may be attributable to the induction of apoptosis via the down-regulation of p-AKT and Bcl-2.

In general, combination therapy is superior to monotherapy in cancer treatment^12^. Early clinical trial results demonstrated that the efficacy of single PI3K inhibitors is limited^45^. Buparlisib, a pan-class PI3K inhibitor, was combined with carboplatin and paclitaxel for patients with advanced solid tumors in a phase I study (ClinicalTrials NCT01297452). The preliminary results indicated that the combination of buparlisib and carboplatin or paclitaxel was well tolerated and exhibited notable effects against tumors^46^. Therefore, we tested the inhibitory effect of econazole in combination with cisplatin in lung cancer cells. We found that the combinations of econazole with cisplatin exhibited additive or synergistic therapeutic efficacy and induced more apoptosis than the single drug. It will be worthwhile to study combinations of econazole with other chemotherapeutic drugs or targeted therapeutic drugs in lung and other cancers.

## Conclusion

Using the structure-based virtual screening tool idock, econazole was identified among a group of FDA-approved drugs as a promising PI3K inhibitor. Econazole exhibited anticancer effects in H661 and A549 lung cancer cells. Furthermore, econazole significantly inhibited downstream target of the PI3K/AKT signaling pathway, the AKT phosphorylation, the expression of Bcl-2, ans induced apoptosis, in H661 and A549 cell lines. The combination of econazole and cisplatin exhibited a stronger effect with respect to the induction of apoptosis than either econazole or cisplatin did alone. More importantly, econazole (50 mg/kg) significantly suppresses A549 tumor growth in nude mice. These results suggest for the first time that econazole is a promising PI3K inhibitor and a candidate anticancer drug for the treatment of lung cancer.

## Materials and Methods

### Molecular docking and compound selection

From the Protein Data Bank (PDB), a total of 14 X-ray crystallographic structures of PI3Kα were identified. Among them, 8 crystal structures of PI3Kα in complex with a large ligand bound at the ATP binding site were selected to use as ensemble docking targets according to the protein conformer selection protocol concluded in a recent study^47^. The PDB codes of the 8 selected structures were as follows: 3HHM, 3ZIM, 4JPS, 4L23, 4L2Y, 4TV3, 4WAF and 4YKN. The PI3Kα structures and the co-crystallized ligands were manually extracted from their corresponding complexes with water molecules removed and then converted from PDB format to PDBQT format with Auto Dock Tools^48^. The cubic search space was placed at the geometrical center of the bound ligand, with the length, width and height set to 30% greater than those of the bound ligand, based on the observation that the geometry of the binding site is often proportional to that of the bound ligand. The search space was then further expanded by 4 Å in all three dimensions to spare sufficient room for the ligand to translate and rotate within the space.

The structures of approved drugs worldwide were obtained from three catalogs of the ZINC database^49^, which are Drug Bank-^50^ and FDA-approved drugs (via DSSTOX), and the NCGC Pharmaceutical Collection (NPC)^51^. These constituted a non-redundant set of 3167 drugs that have been approved for clinical use by the US (FDA), UK (NHS), EU (EMA), Japanese (NHI), and Canadian (HC) authorities. Similarly, these compounds in Mol2 format were also converted to PDBQT format.

The free and open source docking software idock v2.2.1^17^ was used to predict the binding conformations and binding affinities of the 3167 compounds upon docking against the 8 PI3Kα structures. Program settings were tuned to make the conformational searching procedure more exhaustive than the default settings. Specifically, for each protein structure, grid maps of free energy with a fine granularity of 0.08 Å were constructed in parallel. For each compound, 256 Monte Carlo conformational optimization tasks were run in parallel across multiple CPU cores to find the most likely binding conformations.

After docking, up to nine putative conformations were output for each input compound. The docked conformation with the best idock score was selected because it had been previously shown to most closely represent the crystal conformation with a redocking success rate of over 50% on three different benchmarks^16^. The compounds were sorted in ascending order of their predicted binding free energy averaged across the 8 PI3Kα structures. Moreover, the more accurate scoring function RF-Score v3^18^ was used to re-score all of the compounds and thus provide an additional and more reliable estimation of intermolecular binding strength, given the assumption that the compounds were bioactive and correctly docked. Therefore, the top-scoring compounds would be those with both a low idock score (in terms of binding free energy) and a high RF score (in terms of binding affinity). Then, the top-scoring compounds were visually examined using the convenient web-based visualizer iview^19^ in the context of PI3Kα using the X-ray crystal structure of the highest resolution, i.e., PDB code 4JPS. Finally, commercially available compounds were purchased and subsequently validated *in vitro*.

### Chemicals, antibodies, cell lines and cell culture

The selected chemicals (ezetimibe, talniflumate, alvimopan, mizolastine, fupentixol dihydrochloride, doxazosin mesylate, and apixaban), the leading cancer drug econazole and two known PI3K inhibitors (BLY719 and BKM120) were purchased from Copyright J&K Scientific Ltd (Beijing, China).

The lung cancer cell lines NCI-H661 (large cell), A549 (adenocarcinoma), NCI-H520 (squamous) and SK-SEM-1 (squamous) were obtained from the Cell Bank of Kunming Institute of Zoology, Chinese Academy of Sciences. The normal lung bronchial epithelial cell lines BEAS-2B were obtained from Key Laboratory of lung cancer research in Yunnan Province.

NCI-H661 and NCI-H520 cell lines were cultured in RPMI 1640 containing 10% fetal bovine serum (FBS) (Invitrogen, Rockville, Maryland, USA). A549 and SK-SEM-1 cell lines were cultured in DMEM containing 10% FBS (Invitrogen). BEAS-2B cell lines were cultured in F-12 containing 10% FBS (Invitrogen). All media was supplemented with 100 U/ml penicillin and 0.1 mg/ml streptomycin. All cells were cultured at 37 °C in 5% CO_2_.

Antibodies against PI3Kp110α, PI3Kp110δ, total-AKT, phospho-AKT(S473), phospho-AKT(T308), ERK, phospho-ERK, Bcl-2, VEGF, MMP-9, PARP, pro-caspase3, cleaved-caspase3 and β-actin were purchased from Cell Signaling Technology (Massachusetts, USA) and Abcam (San Francisco, USA).

### MTT assays

H661 and A549 cells were seeded in 96-well plates at a density of 1 × 10^5^ cells per well and treated with increasing concentrations of econazole for 24, 36, 48, or 72 hours. The growth inhibitory effect of econazole on lung cancer cells was assessed by the MTT assay. The absorbance was measured at 570 nm with a Synergy 2 microplate reader according to the standard protocol. The IC_50_ values were calculated using SPSS 20.0.

### Western blotting

H661 and A549 cells were seeded in 6-well plates, allowed to attach for 24 hours and treated with media containing econazole at increasing concentrations of 1.25, 2.5, 5, or 10 μM. The cells were harvested 24 hours after incubation. The cells were lysed with RIPA buffer containing 1 mM PMSF and protease inhibitor cocktail at 4 °C for 30 minutes. After centrifugation at 13,000 rpm for 15 minutes, the supernatants were recovered, and the protein concentration was measured using the BCA Protein Assay Kit (Thermo Scientific, Massachusetts, USA). Equal amounts of cell lysates were resolved by 10% SDS-PAGE and transferred onto nitrocellulose membranes (Sigma, Shanghai, China). After blocking with skim milk, the membranes were incubated sequentially with the appropriate diluted primary and secondary antibodies. Proteins were detected using the enhanced chemiluminescence detection system (Amersham, Piscataway, New Jersey, USA). To ensure equal loading of the samples, the membranes were re-probed with an anti-β-actin antibody.

### Flow cytometric analysis of apoptosis

H661 and A549 cells were cultured in 6-well plates. The cells were exposed to econazole, cisplatin, or the combination of econazole and cisplatin for 12 hours. The cells were collected and stained with Annexin V-FITC/propidium iodide (PI) according to the manufacturer’s instructions (Beijing 4 A Biotech Co., Ltd, Beijing, China). The apoptotic cells were analyzed by flow cytometry (BD LSRFortessa™, USA).

### Tumorigenesis assays

Male 5–6 weeks old BALB/C nude mice were purchased from Department of Animal Experiment, Kunming Medical University and were cultured under specific pathogen free conditions. For the xenografted tumor growth assay, A549 cells (1 × 10^6^/0.2 ml PBS) were injected subcutaneously into the right flank of the mice. Seven days after inoculation, the tumors grew to a volume of 70–80 m^3^. The mice were randomly divided into two groups (five mice per group) and injected with Econazole (50 mg/kg in 10%DMSO + 15% castor oil + 75% PBS) by I.P. for 21 days. Tumor volumes were measured every 3–4 days after tumor appearance. Tumor volume was calculated by the equation V = ab^2^/2, where a is the longest axis and b is the shortest axis. The mice were then sacrificed and the tumors were isolated and weighted. This study was approved by the laboratory animal ethics committee of Kunming Medical University.

All methods were performed in accordance with relevant guidelines and regulations.

### Statistical analysis

Data were obtained from at least three different experiments and are expressed as the mean ± SEM (standard error of the mean). Statistical analysis was performed by one-way ANOVA (Analysis Of Variance), and differences were considered statistically significant for p < 0.05. Statistically significant results are marked with an asterisk symbol in the figures.

## References

[1] Dizon DS (2016). Clinical Cancer Advances 2016: Annual Report on Progress Against Cancer From the American Society of Clinical Oncology. J Clin Oncol.

[2] Samuels Y, Waldman T (2010). Oncogenic mutations of PIK3CA in human cancers. Curr Top Microbiol Immunol.

[3] Karakas B, Bachman KE, Park BH (2006). Mutation of the PIK3CA oncogene in human cancers. Br J Cancer.

[4] Dy GK (2013). Epidemiology of Pi3k Pathway Alterations in Patients with Metastatic Non-Small Cell Lung Cancer (Nsclc): Findings from the International Basalt-1 Study. Journal of Thoracic Oncology.

[5] Yamamoto H (2008). PIK3CA mutations and copy number gains in human lung cancers. Cancer Res.

[6] Wang L (2014). PIK3CA mutations frequently coexist with EGFR/KRAS mutations in non-small cell lung cancer and suggest poor prognosis in EGFR/KRAS wildtype subgroup. PLoS One.

[7] Samuels Y (2004). High frequency of mutations of the PIK3CA gene in human cancers. Science.

[8] Dumont AG, Dumont SN, Trent JC (2012). The favorable impact of PIK3CA mutations on survival: an analysis of 2587 patients with breast cancer. Chin J Cancer.

[9] Dienstmann R, Rodon J, Serra V, Tabernero J (2014). Picking the point of inhibition: a comparative review of PI3K/AKT/mTOR pathway inhibitors. Mol Cancer Ther.

[10] Maira SM (2012). Identification and characterization of NVP-BKM120, an orally available pan-class I PI3-kinase inhibitor. Mol Cancer Ther.

[11] Fritsch C (2014). Characterization of the novel and specific PI3Kalpha inhibitor NVP-BYL719 and development of the patient stratification strategy for clinical trials. Mol Cancer Ther.

[12] Vansteenkiste JF (2015). Safety and Efficacy of Buparlisib (BKM120) in Patients with PI3K Pathway-Activated Non-Small Cell Lung Cancer: Results from the Phase II BASALT-1 Study. J Thorac Oncol.

[13] Hu Y (2015). Effects of PI3K inhibitor NVP-BKM120 on overcoming drug resistance and eliminating cancer stem cells in human breast cancer cells. Cell Death Dis.

[14] Allegretti M (2015). The pan-class I phosphatidyl-inositol-3 kinase inhibitor NVP-BKM120 demonstrates anti-leukemic activity in acute myeloid leukemia. Sci Rep.

[15] Shi XN (2015). In Silico Identification and *In Vitro* and *In Vivo* Validation of Anti-Psychotic Drug Fluspirilene as a Potential CDK2 Inhibitor and a Candidate Anti-Cancer Drug. PLoS One.

[16] Li H, Leung KS, Ballester PJ, Wong M (2014). H. istar: a web platform for large-scale protein-ligand docking. PLoS One.

[17] Li, H., Leung, K.-S. & Wong, M.-H. In *Computational Intelligence in Bioinformatics and Computational Biology (CIBCB*), *2012**IEEE Symposium on*. 77–84 (IEEE).

[18] Li HJ, Leung KS, Wong MH, Ballester PJ (2015). Improving AutoDock Vina Using Random Forest: The Growing Accuracy of Binding Affinity Prediction by the Effective Exploitation of Larger Data Sets. Mol Inform.

[19] Li H, Leung KS, Nakane T, Wong M (2014). H. iview: an interactive WebGL visualizer for protein-ligand complex. BMC Bioinformatics.

[20] Rosell R (2012). Erlotinib versus standard chemotherapy as first-line treatment for European patients with advanced EGFR mutation-positive non-small-cell lung cancer (EURTAC): a multicentre, open-label, randomised phase 3 trial. Lancet Oncology.

[21] Kwak EL (2010). Anaplastic Lymphoma Kinase Inhibition in Non-Small-Cell Lung Cancer. New Engl J Med.

[22] Oxnard GR, Binder A, Janne PA (2013). New targetable oncogenes in non-small-cell lung cancer. J Clin Oncol.

[23] Scheffler M (2015). PIK3CA mutations in non-small cell lung cancer (NSCLC): genetic heterogeneity, prognostic impact and incidence of prior malignancies. Oncotarget.

[24] Vanhaesebroeck B, Guillermet-Guibert J, Graupera M, Bilanges B (2010). The emerging mechanisms of isoform-specific PI3K signalling. Nat Rev Mol Cell Bio.

[25] Dunlap J (2010). Phosphatidylinositol-3-kinase and AKT1 mutations occur early in breast carcinoma. Breast Cancer Res Tr.

[26] Najafov A, Shpiro N, Alessi DR (2012). Akt is efficiently activated by PIF-pocket- and PtdIns(3,4,5)P-3-dependent mechanisms leading to resistance to PDK1 inhibitors. Biochemical Journal.

[27] Schuurbiers OC (2009). The PI3-K/AKT-pathway and radiation resistance mechanisms in non-small cell lung cancer. J Thorac Oncol.

[28] Bao R (2009). Targeting heat shock protein 90 with CUDC-305 overcomes erlotinib resistance in non-small cell lung cancer. Mol Cancer Ther.

[29] Zou ZQ (2009). A novel dual PI3Kalpha/mTOR inhibitor PI-103 with high antitumor activity in non-small cell lung cancer cells. Int J Mol Med.

[30] Ueda Y (2009). Synergistic cell growth inhibition by the combination of amrubicin and Akt-suppressing tyrosine kinase inhibitors in small cell lung cancer cells: implication of c-Src and its inhibitor. Int J Oncol.

[31] Curtin NJ, Szabo C (2013). Therapeutic applications of PARP inhibitors: anticancer therapy and beyond. Mol Aspects Med.

[32] Magesh V (2009). Ocimum sanctum induces apoptosis in A549 lung cancer cells and suppresses the *in vivo* growth of Lewis lung carcinoma cells. Phytother Res.

[33] Massacesi C (2016). PI3K inhibitors as new cancer therapeutics: implications for clinical trial design. Oncotargets Ther.

[34] Marcondes MC, Sola-Penna M, Zancan P (2010). Clotrimazole potentiates the inhibitory effects of ATP on the key glycolytic enzyme 6-phosphofructo-1-kinase. Arch Biochem Biophys.

[35] Sun J (2014). Econazole Nitrate Induces Apoptosis in MCF-7 Cells via Mitochondrial and Caspase Pathways. Iran J Pharm Res.

[36] Chi CC, Chou CT, Liang WZ, Jan CR (2014). Effect of the pesticide, deltamethrin, on Ca2+ signaling and apoptosis in OC2 human oral cancer cells. Drug Chem Toxicol.

[37] Cogswell S, Berger S, Waterhouse D, Bally MB, Wasan EK (2006). A parenteral econazole formulation using a novel micelle-to-liposome transfer method: *in vitro* characterization and tumor growth delay in a breast cancer xenograft model. Pharm Res.

[38] Najid A, Ratinaud MH (1991). Comparative studies of steroidogenesis inhibitors (econazole, ketoconazole) on human breast cancer MCF-7 cell proliferation by growth experiments, thymidine incorporation and flow cytometric DNA analysis. Tumori.

[39] Huang JK (2005). Effects of econazole on Ca2+ levels in and the growth of human prostate cancer PC3 cells. Clin Exp Pharmacol Physiol.

[40] Ho YS (2005). Molecular mechanisms of econazole-induced toxicity on human colon cancer cells: G0/G1 cell cycle arrest and caspase 8-independent apoptotic signaling pathways. Food Chem Toxicol.

[41] Liu R (2014). Itraconazole suppresses the growth of glioblastoma through induction of autophagy: involvement of abnormal cholesterol trafficking. Autophagy.

[42] Tsubamoto H (2017). Itraconazole Inhibits AKT/mTOR Signaling and Proliferation in Endometrial Cancer Cells. Anticancer Res.

[43] Liang G (2017). Itraconazole exerts its anti-melanoma effect by suppressing Hedgehog, Wnt, and PI3K/mTOR signaling pathways. Oncotarget.

[44] Kim SY, Yoo SJ, Ronnett GV, Kim EK, Moon C (2015). Odorant Stimulation Promotes Survival of Rodent Olfactory Receptor Neurons via PI3K/Akt Activation and Bcl-2 Expression. Mol Cells.

[45] Okkenhaug K, Graupera M, Vanhaesebroeck B (2016). Targeting PI3K in Cancer: Impact on Tumor Cells, Their Protective Stroma, Angiogenesis, and Immunotherapy. Cancer Discovery.

[46] Hyman DM (2015). Parallel phase Ib studies of two schedules of buparlisib (BKM120) plus carboplatin and paclitaxel (q21 days or q28 days) for patients with advanced solid tumors. Cancer Chemoth Pharm.

[47] Huang Z, Wong CF (2016). Inexpensive Method for Selecting Receptor Structures for Virtual Screening. J Chem Inf Model.

[48] Morris GM (2009). AutoDock4 and AutoDockTools4: Automated docking with selective receptor flexibility. J Comput Chem.

[49] Irwin JJ, Sterling T, Mysinger MM, Bolstad ES, Coleman RG (2012). ZINC: a free tool to discover chemistry for biology. J Chem Inf Model.

[50] Law V (2014). DrugBank 4.0: shedding new light on drug metabolism. Nucleic Acids Res.

[51] Huang R (2011). The NCGC pharmaceutical collection: a comprehensive resource of clinically approved drugs enabling repurposing and chemical genomics. Sci Transl Med.

